# Draft genomic sequence of *Nereida ignava* CECT 5292^T^, a marine bacterium of the family *Rhodobacteraceae*

**DOI:** 10.1186/s40793-016-0141-2

**Published:** 2016-02-29

**Authors:** David R. Arahal, María J. Pujalte, Lidia Rodrigo-Torres

**Affiliations:** Departamento de Microbiología y Ecología and Colección Española de Cultivos Tipo (CECT), Universitat de València, Burjassot, 46100 Valencia Spain

**Keywords:** *Nereida ignava*, *Rhodobacteraceae*, Mediterranean Sea, Strictly aerobic, Slightly halophilic, Marine bacteria

## Abstract

*Nereida ignava* strain 2SM4^T^ (= CECT 5292^T^ = DSM 16309^T^ = CIP 108404^T^ = CCUG 49433^T^) is a marine bacterium belonging to the *Roseobacter* group of the family *Rhodobacteraceae* within the class *Alphaproteobacteria*. The strain was isolated from sea water surrounding cultivated oysters 2–3 miles off the Mediterranean coast near Valencia (Spain) and was phylogenetically related to uncultured clones of gall symbiont bacteria of some species of *Prionitis* alga. Here we describe the genome sequence and annotation of this organism, the type strain of the single species of this genus. The genome comprised 2,888,349 bp, 2,872 protein-coding genes and 52 RNA genes. The annotation revealed the capacity to produce bacteriocins, vitamins and auxins. Besides, it contained sulfur cycling related genes.

## Introduction

*Nereida* is a genus of the *Roseobacter* group, within the family *Rhodobacteraceae*, order *Rhodobacterales**,* Class *Alphaproteobacteria*, so far containing only one species, *Nereida ignava* [1]. At the time of writing there are 197 genome assemblies from members of the family *Rhodobacteraceae* available at NCBI and only 52 of the 253 type strains within the family had a genome project, based on MEP. Strain 2SM4^T^, isolated from Mediterranean coast, served to describe the species so it is the type strain.

Members of the *Roseobacter* group are bacteria linked to a wide variety of marine environments and types of metabolism, playing an important role in carbon, sulfur and nitrogen cycling [2]. Some bacteria belonging to this group were described as epiphytes or symbionts of marine organisms. Genome analysis and culture experiments have revealed mechanisms by which members of this group may associate and interact with phytoplankton and other eukaryotes [3–5].

The 16S rRNA gene sequence phylogeny placed this strain in the same cluster as uncultured gall symbionts of the red algal genus *Prionitis* [1]. Gall formation in this alga was reported to be induced by bacteria phylogenetically related to the *Roseobacter* group [6, 7]. It is known that this relationship is species specific and coevolution exists between host and bacteria [8]. Currently, the established species more closely related to *N. ignava* 2SM4^T^ are *Pseudooctadecabacter jejudonensis* and *Lentibacter algarum* with which it shares 95.88 % and 95.61 % 16S rRNA gene sequence similarity, respectively (EzTaxon [9]).

Here, we present a description of the draft genome sequence and annotation of *N. ignava* 2SM4^T^ type strain. The genomic insights indicate the genetic potential for the synthesis of vitamins, auxin and secondary metabolite production, bacteriochlorophyll *a*, photosynthetic reaction centers, photorespiration and utilization of algae derived compounds, which may explain the close relationship to the gall symbiont bacteria.

## Organism information

### Classification and features

**Fig. 1:**
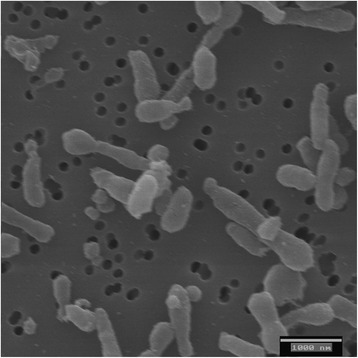
Scanning electron micrograph showing the characteristic variations in cell morphology of *N.* *ignava* 2SM4^T^, from coccoid cells to elongated rods, including many tear-shaped cells

*N. ignava* 2SM4^T^ was obtained from Mediterranean Sea water 2–3 miles off the Spanish coast near Valencia, in the surrounding of cultivated oysters [1]. It is a Gram-negative, slightly halophilic, non-pigmented, strictly aerobic, non-motile, mesophilic, chemo-organotrophic bacterium (Table 1). Cells are coccoid to elongate rods, most of them are tear-shaped and show polar budding (Fig. 1). They measure 0.2–0.3 μm in width by 1–3 μm in length. Mesophilic. Neither gas vesicles nor poly-β-hydroxybutyrate accumulation has been observed. Does not ferment carbohydrates and is unable to reduce nitrate to nitrite or N_2_ [1].

**Table 1:** Classification and general features of *Nereida ignava* strain 2SM4^T^in accordance with the MIGS recommendations [38] published by the Genome Standards Consortium [39]

MIGS ID	Property	Term	Evidence code^a^
	Classification	Domain *Bacteria*	TAS [40]
		Phylum *Proteobacteria*	TAS [41]
		Class *Alphaproteobacteria*	TAS [42]
		Order *Rhodobacterales*	TAS [43]
		Family *Rhodobacteraceae*	TAS [44]
		Genus *Nereida*	TAS [1]
		Species *Nereida ignava*	TAS [1]
		(Type) strain: 2SM4^T^(= CECT 5292^T^ = DSM 16309^T^ = CIP 108404^T^ = CCUG 49433^T^)	
	Gram stain	Negative	TAS [1]
	Cell shape	Coccoid to elongated rods	TAS [1]
	Motility	Non motile	TAS [1]
	Sporulation	Not reported	NAS
	Temperature range	13–28 °C	TAS [1]
	Optimum temperature	24–26 °C	TAS [1]
	pH range; Optimum	Neutral	TAS [1]
	Carbon source	Maltose, succinate, fumarate, malate, lactate, sarcosine	TAS [1]
MIGS-6	Habitat	Marine	TAS [1]
MIGS-6.3	Salinity	3,5 %	TAS [1]
MIGS-22	Oxygen requirement	Aerobic	TAS [1]
MIGS-15	Biotic relationship	Free-living	TAS [1]
MIGS-14	Pathogenicity	Not reported	NAS
MIGS-4	Geographic location	Vinaroz, Spain	TAS [1]
MIGS-5	Sample collection	1990	TAS [1]
MIGS-4.1	Latitude	40,46	TAS [1]
MIGS-4.2	Longitude	0.50	TAS [1]
MIGS-4.4	Altitude	0 m	TAS [1]

^a^ Evidence codes - *IDA* Inferred from Direct Assay, *TAS* Traceable Author Statement (i.e., a direct report exists in the literature), *NAS* Non-traceable Author Statement (i.e., not directly observed for the living, isolated sample, but based on a generally accepted property for the species, or anecdotal evidence). These evidence codes are from the Gene Ontology project [45]

**Fig. 2:**
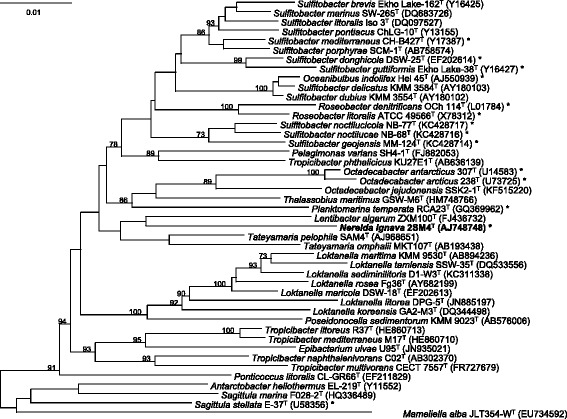
Phylogenetic tree highlighting the position of *N. ignava* 2SM4^T^ (bold) relative to other strains of the *Roseobacter* group. SSU rRNA gene sequences were aligned with version 121 of the ‘All-Species Living Tree’ project SSU rRNA gene database [35] using the ARB software package [36]. The phylogeny was constructed from nearly full-length gene sequences using the neighbor-joining method [37] within ARB, filtered to exclude alignment positions that contained gaps or ambiguous nucleotides in any of the sequences included in the tree. Nodes with bootstrap support above 70 % are indicated. An asterisk stands for strains (including equivalent designations) that have a genome sequence in the NCBI Assemble database. A variety of *Archaea* were used as outgroups

Comparative analysis of the 16S rRNA gene sequence to species belonging to the *Roseobacter* group (Fig. 2) indicates that *N. ignava* 2SM4^T^ most related bacterium is *L. algarum* ZXM100^T^ with which it forms a cluster. Searching for 16S rRNA gene sequence similarities in EzTaxon [9], 95.88 % similarity was found with *P. jejudonensis* SSK2-1^T^and 95.61 % with *L. algarum* ZXM100^T^.

#### Chemotaxonomic data

The predominant fatty acids are C18:1*ω*7*c* (81.4 %), 18:0 (6.0 %) and 16:0 (5.0 %) followed by 11-methyl 18:1*ω*7*c* (3.3 %), 10:0 3-OH (1.9 %), 19 : 1*ω6c* or 19 : 0 cyclo (1.5 %) and 20:1*ω*7*c* (0.8 %) [1]. The fatty acid profile confirmed the affiliation to the *Roseobacter* group.

## Genome sequencing information

### Genome project history

*N. ignava* 2SM4^T^ was selected for genome sequencing among a larger set of marine strains isolated and characterized by our research group during the last three decades, based on its phylogenetic position and its biological significance. The draft genome sequencing project was registered in the European Nucleotide Archive database under accession number ERP010009, together with the sequence read archives (ERR833218), and annotated contigs CVQV01000001-CVQV01000069. Table 2 presents the main project information and its compliance with MIGS version 2.0.

**Table 2:** Project information

MIGS ID	Property	Term
MIGS 31	Finishing quality	Draft
MIGS-28	Libraries used	One Illumina Miseq paired end library
MIGS 29	Sequencing platforms	Illumina Miseq
MIGS 31.2	Fold coverage	170x
MIGS 30	Assemblers	MIRA v4.0
MIGS 32	Gene calling method	Prodigal v2.60 in Prokka
	Locus Tag	NIG5292
	Genbank ID	CVQV00000000
	GenBank Date of Release	25-May-2015
	GOLD ID	-
	BIOPROJECT	PRJEB8965
MIGS 13	Source Material Identifier	CECT 5292
	Project relevance	Type strain

### Growth conditions and genomic DNA preparation

*N. ignava* 2SM4^T^ was cultured in marine agar (MA; Difco) at 26 °C under aerobic conditions during three days. Genomic DNA was isolated using Real Pure Spin kit (Durviz) following the standard protocol recommended by the manufacturer. The integrity of the extracted DNA was checked by visualization in a 2.0 % agarose gel electrophoresis. Its purity and quantity was checked by measuring the absorbance at 260 and 280 nm with a spectrophotometer Nanodrop2000c (Thermo Scientific) and calculating the ratio A260/A280. A total of 10.6 μg of DNA were obtained.

### Genome sequencing and assembly

The genome of *N. ignava* 2SM4^T^ was sequenced at Central Service of Support to Experimental Research (SCSIE) of the University of Valencia (Valencia, Spain) using an Illumina Miseq technology with 2 x 250 paired-end reads. The sequencing experiment yielded 1,991,958 reads totaling 495,586,123 bases which accounts for an approximate 170x sequencing coverage. The Illumina reads were analyzed for quality control using FASTQC, a common quality control tool developed by Babraham Bioinformatics to check raw sequencing data, which is wrapped in Galaxy Orione Server [10]. After filtering, the remaining reads were assembled using MIRA v4.0 de novo assembler [11] incorporated as a tool in Galaxy Orione Server. The final draft assembly contained 69 contigs larger than 1,000 nucleotides and had a total size of 2,888,349 bp. An average 77x coverage depth was accomplished.

### Genome annotation

The draft genome was annotated using Prokka [12], an open source software tool, within Galaxy Orione Server, and using RAST v2.0 (Rapid Annotation using Subsystem Technology) [13]. CRISPR repeats were examined by CRISPR Finder [14]. Signal peptides were searched using Signal P 4.1 Server [15]. Transmembrane helix domains were predicted through TMHMM server v.2.0 [16]. Protein coding genes were analyzed for COG functional annotation using WebMGA server [17]. Secondary metabolites were predicted using antiSMASH 2.0 [18]. Pfam domains were predicted using NCBI Batch CD-Search Tool [19] using default parameters.

## Genome properties

The draft genome consists of 69 contigs containing 2,888,349 bp. The G + C content, determined by RAST, was 54 %. From a total of 2,924 genes predicted with Prokka, 2,872 were protein-coding genes and 52 were RNA genes (45 tRNA and 7 rRNA genes). 76.6 % protein coding genes were assigned to putative functions and 23.4 % remained as hypothetical proteins (Table 3). Among protein-coding genes, 48 % were assigned to subsystems using RAST. The distribution of genes into COG functional categories is shown in Table 4.

**Table 3:** Genome statistics (Prokka)

Attribute	Value	% of total
Genome size (bp)	2,8883,49	100.0
DNA coding (bp)	2,567,900	88.9
DNA G + C (bp)	1,559,709	54.0
DNA scaffolds	69	100.0
Total genes	2,924	100.0
Protein coding genes	2,872	98.2
RNA genes	52	1.8
Pseudo genes	-	-
Genes in internal clusters	-	-
Genes with function prediction	2,249	76.9
Genes assigned to COGs	2,207	75.5
Genes with Pfam domains	2,069	70.8
Genes with signal peptides	208	7.1
Genes with transmembrane helices	619	21.6
CRISPR repeats	0	0.0

**Table 4:** Number of genes associated with general COG functional categories

Code	Value	%age	Description
J	159	5.4	Translation, ribosomal structure and biogenesis
A	0	0.0	RNA processing and modification
K	151	5.2	Transcription
L	158	5.4	Replication, recombination and repair
B	2	0.0	Chromatin structure and dynamics
D	26	0.9	Cell cycle control, Cell division, chromosome partitioning
V	26	0.9	Defense mechanisms
T	104	3.6	Signal transduction mechanisms
M	127	4.3	Cell wall/membrane biogenesis
N	49	1.7	Cell motility
U	45	1.5	Intracellular trafficking and secretion
O	110	3.8	Posttranslational modification, protein turnover, chaperones
C	173	5.9	Energy production and conversion
G	178	6.1	Carbohydrate transport and metabolism
E	261	8.9	Amino acid transport and metabolism
F	64	2.2	Nucleotide transport and metabolism
H	129	4.4	Coenzyme transport and metabolism
I	101	3.5	Lipid transport and metabolism
P	106	3.6	Inorganic ion transport and metabolism
Q	73	2.5	Secondary metabolites biosynthesis, transport and catabolism
R	284	9.7	General function prediction only
S	205	7.0	Function unknown
-	717	24.5	Not in COGs

The total is based on the total number of protein coding genes in the genome

## Insights from the genome sequence

The genome of *N. ignava* 2SM4^T^ is one of the smallest ones described to date among members of the *Roseobacter* group [20], but its size is in accordance with previous reports about its possible symbiotic character [21]. It harbors all the gene repertoire of the tricarboxylic acid cycle, Entner-Doudoroff route and the Pentose-Phosphate pathway. However the glycolysis pathway, lacking phosphofructokinase I, is incomplete. Similarly, the Glyoxylate cycle pathway is incomplete as isocitrate lyase is not annotated. Many serine cycle methylotrophs do not contain isocitrate lyase and transform Acetyl-CoA into glyoxylate via a route which implies butyryl-CoA and propionyl-CoA intermediates (coming from PHB degradation), ethylmalonyl CoA pathway [22]. This genome contains most of the genes acting in this route, including glycine hydroxymethyl transferase which introduces, into serine cycle, methylene tetrahydrofolate coming from methanol. The presence of these genes suggests *N. ignava* 2SM4^T^ can use C1 and C2 compounds as carbon source.

Ammonium assimilation is possible thanks to the presence of genes involved in transport and assimilation such as *amt*, *glnA*, *glnB*, *glnG, glnL, glnQ*, *glnM*, *gltB* and *gltD*. A *nas**A* gene coding for a nitrate transporter was predicted but no nitrate or nitrite reductases were found. Five genes involved in nitrogen fixation were annotated: *fixK,* coding for a nitrogen fixation regulatory protein, *nifS, nifU* coding for nitrogenase metallic centre biosynthesis, *nif-H1*, coding for a nitrogenase iron protein (*nifH* encodes nitrogenase reductase, one of the two components of nitrogenase) and *ntrC* gene, coding for NtrC protein, the regulator that switch on/off the nitrogen fixation through ammonium availability inside the cell; however, genes *nifDK* coding for nitrogenase were not found. This fact suggests some possible scenarios: a loss of the potential nitrogen fixation ability, a possible current event of gene gaining still uncompleted or the absence of the complete gene cluster due to the condition of draft genome. A *ydjA* coding putative NAD(P)H nitroreductase gene was predicted suggesting reduction of nitro group containing compounds. Reduced organic nitrogen compounds appear to be used, as many nitrogenated substances (taurine, betaine, aminoacids, urea) transporters and utilization genes were annotated. Reaffirming the findings of Chen (2012) [23], this genome presented two trimethylamine methyltransferase family protein genes, revealing the potential capability of using methylated amines as nitrogen source.

Phosphate ions uptake can be done through high affinity phosphate transporters (*pstA, B, C, S*) and regulatory genes (*phoB, P, R, U*) and alkaline phosphatase. The genome also contains a low affinity phosphate transporter (*pitA*) and 13 *phn* genes for phosphonate utilization.

## Extended insights

In order to gain knowledge about the biology of *N. ignava* 2SM4^T^, we explored its genome searching for diverse metabolic, physiological and structural characteristics described in other roseobacters and the outcome of these searches is commented in the following paragraphs.

*N. ignava* 2SM4^T^ draft genome encodes all necessary genes for assimilatory sulfate reduction (*cys* genes), except *cysH* gene coding for phosphoadenosine phosphosulfate (PAPS) reductase, in view of this, the ability of performing this activity cannot be concluded. DMSP is also metabolized. DMSP is a sulfur organic compound produced by phytoplankton for osmoregulation and is a carbon and sulfur source in marine environment for bacterioplankton [3]. There are two pathways to use DMSP: cleavage and demethylation. Cleavage pathway releases DMS which is a climate-active gas important in atmospheric-cooling. Demethylation pathway allows sulfur assimilation. Four genes of this route (*dmdA*_1, *dmdA*_2, *B*, *C*) were predicted but no cleavage pathway genes were found. Thus, in the absence of the ability to perform a complete nitrate and sulfate assimilatory reductions, the bacterium seems mostly compelled to obtain N and S from organic sources. We also observed that siderophore production is also absent, so the species is closely dependent of organic matter and reduced forms for its mineral nutrition.

Many marine environments are known because of the organic nutrients scarceness and this is why many marine heterotrophic bacteria develop strategies to obtain energy from different sources. One of the mechanisms is using reduced inorganic compounds. In this sense, the complete set of *sox* genes (*soxRSVWXYZABCDEF*) is present in the annotated genome, conferring the ability to oxidize reduced inorganic sulfur compounds such as thiosulfate, a very common compound in seawater and utilized by other members of *Roseobacter* group [24]. Another mechanism is by oxidizing CO to CO_2_ by means of carbon monoxide dehydrogenase, encoded by *coxL* form I and II. These genes were not annotated in the genome indicating this strain is not able to use this source of energy. Phototrophy appears to be a way to increase efficiency of heterotrophic growth for many members of *Roseobacter* group [25]. Photosynthetic genes were detected comprising a large photosynthetic gene cluster as in other *Roseobacter* strains [26]: bacteriochlorophyll *a* and carotenoid genes (*bch* and *crt*), photosynthetic reaction centre and light-harvesting antenna complex genes (*puf* and *puh*) and a sensor of blue light using FAD (BLUF domain). BLUF sensor domains are hypothesized to be involved in light-dependent regulation of the photosynthesis operon. The ability to synthetize bchl *a* and carotenoids was unexpected, as the strain has been unpigmented in every growth condition assayed in our lab. Chemotaxis and chemoreceptor genes were also checked and the genome harbors 12 genes involved in both activities. Light sensing for phototrophy is supported by 3 genes encoding blue light activated proteins and the sensor for blue light mentioned. However, but not unexpected, rubisco genes were absent.

The genome of *N. ignava* 2SM4^T^ shows several genes that may be related to its phylogenetic position close to algal symbionts [1]. As previously reported [5, 27], members of *Roseobacter* group enhance phytoplankton colonization by producing antibiotics that prevent the growth of other bacteria and promote that of phytoplankton by synthetizing auxin and vitamins. Genes related to these functions were also located in genomes of other alphaproteobacteria associated with primary producers [28]. According to antiSMASH v2.0 program, this genome contains two terpene gene clusters, one bacteriocin gene cluster, one microcin gene cluster and a homoserine lactone gene cluster. In addition, seven genes were identified as belonging to Colicin V and Bacteriocin production cluster, while auxin biosynthesis, transport and degradation and group B (B1, B2, B3, B6, B9, B12) vitamins biosynthesis genes, that were also annotated, suggest this strain could have a biotechnological application.

Drug/metabolite transporters superfamily (DMTs) [29] are a group of transporters found ubiquitously and also in *Roseobacter* group [30]. They use ion gradients instead of ATP, which may be useful in marine environment, and in roseobacters, they average 30 genes per genome compared to 16 for other alphaproteobacteria. However, in the genome of *N. ignava* 2SM4^T^ only 11 genes belonging to this superfamily and one putative DMT superfamily transporter were found. This can be explained because of the small size of the genome. Moreover, 6 multidrug export proteins genes (*mepA*, *mepA*_2, *mdtA*, *mdtK*, two putative multidrug export ATP-binding/permease) and resistance proteins to antibiotics such as tetracycline, spectinomycin, chloramphenicol and camphor were also predicted. In addition, the genome of strain 2SM4^T^ encodes 6 beta-lactamase genes and a complete mechanism for fluoroquinolones resistance. Other genes encoding for copper homeostasis enzymes, mercury reductase were also predicted. These genes confer to this bacterium a strong potential in competing with other microorganisms and tolerating heavy metals.

A reduced number of ABC, TRAP and DMT transporters was related to small size of the genome in recent studies [20]. ABC transporters use energy of ATP breakdown to move a wide range of substrates across membranes while TRAP transporters use ion gradients instead. 140 ABC transporters were predicted using RAST annotation pipeline, which is in contrast with the average value of 279 genes contained in genomes of *Roseobacter* group according to Durham et al. [20]. TRAP transporters were also predicted below the average number in the *Roseobacter* group, 34 versus 60. Again this trend is in agreement with the reduced genome size (and may be related to the narrow range of substrates that the strain uses as sole carbon and energy sources).

The ability of synthetizing virulence factors was explored and an *apxIB* gene coding a RTX-I toxin determinant B together with two genes coding for Type I Secretion System proteins (PrsE_1 and TolC), a plasmid hemolysin genes (*hlyA*_2) and a hemolysin secretion protein (*hlyD*) were annotated contiguously in the genome suggesting they form an operon. A similar structure but without the RTX-I toxin gene was found in the genome in a different location while 3 hemolysin, a leukotoxin and a ribosome associated toxin RatA genes were located dispersedly in different parts of the genome. These findings support the idea that roseobacters frequently interact with neighboring cells to increase the possibilities to access resources.

Quorum sensing is a process of cell-to-cell communication between related cells, which is dependent on the detection of a certain detected population threshold value. Small molecules act as autoinducers when the concentration of these molecules reaches a threshold value and then they activate gene expression to coordinate population behavior. This is especially important in bacteria colonizing or infecting hosts as this determines its success. No *luxI* genes were predicted but 3 *luxR* family proteins were annotated by RAST and also found in Prokka under regulatory protein designation. On the bacterial chromosome, *luxR* genes are usually found next or near to a *luxI* gene encoding the AHL signal synthase, but recently *luxR* alone was examined through bacterial genomes [31]. This “loneliness” appears to be quite common suggesting they may either respond to internal AHL signals produced by a non-adjacent *luxI* in the chromosome or can respond to exogenous signals. Two autoinducer 2 sensor kinase/phosphatase *luxQ* genes were predicted.

Genes encoding signal transduction proteins are present in *Roseobacter* group genomes as a mechanism to respond to environmental stimuli [30]. Amino acid metabolism seems important in this strain as eight Lrp genes involved in this metabolism were predicted, exceeding in number and variety those involved in carbohydrate utilization. Eight MarR genes are also found, involved in antibiotic resistance and stress response, and GntR and IclR encoding repressors of gluconate utilization and acetate operon respectively were also encoded.

Although no swarming or motility *via* flagella were observed in cells [1], a large number of flagella machinery genes and a swarming protein gene were annotated: 22 *fli* genes, 15 *flg* genes, 2 *flh* genes, a flagellar assembly protein H gene, a Ylx H flagellum site-determining protein gene, a flagellar basal body rod modification protein, a quorum sensing regulator protein F gene and a swarming motility protein SwrC gene. The majority of these genes were located contiguously in the genome. In line with this, capsule and biofilm formation genes were also examined. The genome contains 3 capsule polysaccharide biosynthesis protein genes, two Vi polysaccharide export inner membrane protein genes, vexB and vexD, two acid polysialic transport protein and other polysaccharide export protein genes. Colonies were not reported to have mucosal appearance [1], but these genes suggest cells have the ability to synthetize some capsular components which expression may be induced under determined environmental circumstances. Moreover, two biofilm growth associated repressor genes and an intercellular adhesion protein R (icaR gene) were found. Fimbriae and pili genes were also encoded in this bacterium draft genome: two Flp pilus assembly protein TadG, a helicase Rec Q associated with Flp pilus assembly and type IV pilus biogenesis protein PilW and conjugation transfer ATPase VirB4 family. Two genes associated with curli fimbriae and cellulose extracellular matrix formation in enterobacteria [32], csgA and bcsA gene, were also predicted in the genome as well as two succinoglycan biosynthesis proteins exoI. Thus, adhesion genes may support the ability of communication between cells and attaching to surfaces as a symbiont bacterium if this biological condition is confirmed.

CRISPR repeats were not detected, however, three type III restriction-modification system proteins, methylase, helicase and one putative hydrolase, were encoded, indicating that this strain has a putative mechanism of defense from foreign nucleic acids.

Plasmids are present in many members of the *Roseobacter* group [33]. Further study must be done to close the genome sequence and discover plasmids encoded by *N. ignava* 2SM4^T^. However, there is certain evidence suggesting plasmid presence. As previously remarked, two plasmid hemolysins were annotated, but also a plasmid oligopeptidase F, whose position is close to a type IV pilus biogenesis/stability protein PilW, two C-terminal domains of phage/plasmid primase belonging to P4 family and a type IV secretion/conjugal transfer ATPase belonging to VirB4 family. A postsegregational killing system (PSK) can be deducted by the codification of an antitoxin of toxin-antitoxin stability system N-terminal, but no closely toxin gene was found.

Cell division genes (*fts*) and rod shape-determining proteins genes (*rodA, mreB, mreC*) were encoded in the genome, however no *min* genes [34], determining the central axis in cell division, were found. This absence may be related with the budding division shown by *N. ignava* and other members of the *Roseobacter* group; in fact, *min* genes seem to be rare in the *Roseobacter* genomes examined so far.

## Conclusions

The draft genome of *N. ignava* 2SM4^T^ was described. This strain was selected for genome sequencing among a larger set of marine strains isolated and characterized by our research group during the last three decades, due to its distant evolutionary relationship with other members of the *Rhodobacteraceae* family, to provide reference material as a unique member of the genus and species and to study its biological potential. The genes annotated revealed an important potential role in carbon, nitrogen and sulfur cycling in marine environment. A mixotrophic growth could be possible thanks to the presence of the adequate gene repertoire. The small size of the genome and auxin, vitamins and secondary metabolites production genes may explain its phylogenetic relationship with gall symbiont bacteria and display a potential use for biotechnological purposes.

## Abbreviations

ABC: ATP-binding cassette
COG: Clusters of Orthologous Groups
DMSP: Dimethylsulfoniopropionate
MEP: Microbial earth project
PHB: Polyhydroxybutyrate.
TRAP: Tripartite ATP-independent Periplasmic
